# Gait and Plantar Pressure Analysis in Charcot Neuroarthropathy: A Clinic‐Based Biomechanical Study

**DOI:** 10.1002/jfa2.70224

**Published:** 2026-10-12

**Authors:** Irene M. Laleye, Zachary A. Koroneos, Akhil Bolisetti, Carlie Hernjak, Titus Richardson, Shelby Alwine, Parker Fox, Ashlee MacDonald, Michael A. Aynardi

**Affiliations:** ^1^ Penn State College of Medicine Department of Orthopaedics and Rehabilitation The Pennsylvania State University Hershey Pennsylvania USA; ^2^ Penn State University Department of Mechanical Engineering University Park PA USA

**Keywords:** charcot neuroarthropathy, diabetes mellitus, gait analysis, neuropathy, plantar pressure

## Abstract

**Introduction:**

Charcot Neuroarthropathy (CN) is a progressive joint disorder resulting from peripheral neuropathy and repetitive microtrauma. Management prioritizes offloading to prevent deformity, ulceration, infection, and amputation. While three‐dimensional gait analysis can be useful for disease staging and guiding management, its cost and limited accessibility limit routine clinical use. This study evaluated plantar pressure and two‐dimensional gait kinematics in CN patients using a relatively low‐cost clinic‐based approach.

**Methods:**

Following institutional review board approval, this cross‐sectional observational study was conducted at a single institution between July and December 2024. Nine patients with clinically diagnosed unilateral CN and 11 Healthy controls were enrolled. Participants walked at 1.0 mph on a treadmill wearing diabetic slippers equipped with insole pressure sensors and markers placed on standardized lower‐extremity landmarks. Peak plantar pressures were calculated for each step and averaged within predefined regions of interest (forefoot, metatarsals, midfoot, and hindfoot); median region‐specific values were used for group comparisons. The hip, knee, and ankle joint range of motion (ROM) were also assessed. Comparisons were made between affected and contralateral CN limbs, and between each of these limbs and Healthy controls.

**Results:**

Affected limbs demonstrated significantly higher midfoot and significantly lower forefoot, hindfoot, and metatarsal plantar pressures than Healthy controls (Benjamini‐Hochberg adjusted *p* < 0.05 for all four regions). Knee ROM was significantly reduced in both affected and contralateral limbs compared with Healthy controls (adjusted *p* < 0.05 for both), whereas hip and ankle ROM did not differ significantly across groups. No affected‐versus‐contralateral differences survived correction for multiple comparisons in either plantar pressure or kinematic outcomes.

**Conclusions:**

CN participants exhibited reduced knee ROM and altered regional plantar pressures consistent with midfoot collapse, with reduced knee ROM also evident in the contralateral limb, supporting bilateral involvement in CN. These findings support the utility of clinic‐based integrated gait analysis as an adjunctive tool for characterizing CN and informing clinical management.

## 1 Introduction

Charcot neuroarthropathy (CN) is a severe complication of peripheral neuropathy that leads to chronic inflammation and progressive destruction of the bones, joints, and soft tissues of the foot [1, 2]. Diabetes is the leading cause of peripheral neuropathy in developed countries, with CN affecting an estimated 35% of patients with diabetic neuropathy [1, 3]. As CN progresses, the resulting foot deformities contribute to elevated plantar pressures and increased risk of ulceration. Untreated, these changes can lead to serious morbidity, including infection and amputation [1, 4, 5].

The foot and ankle function as an integrated biomechanical unit, with the midfoot being the most commonly affected site in CN, particularly the Lisfranc and Chopart [3, 4, 6]. The Lisfranc joint serves as the structural keystone of the transverse arch, while the Chopart joint enables the midfoot to adapt to uneven terrain and provides rigidity during toe‐off. The Lisfranc joint also plays a critical role in the windlass mechanism, a dynamic interaction between the midfoot, forefoot, hindfoot, and plantar fascia that transforms the foot into a rigid lever during toe‐off [6, 7]. CN's pathophysiology involves a hyperemia‐driven inflammatory process causing osteoclastic bone resorption and periarticular ligamentous laxity, occurring alongside plantar aponeurosis dysfunction. Rupture or structural failure of the plantar aponeurosis has been documented in CN affected feet, further supporting its role as an integral component of the biomechanical changes associated with CN [1, 2, 7]. Damage to these structures destabilizes the foot, disrupts load‐bearing capacity, and alters gait mechanics [2, 4]. The structural complexity of the foot and diagnostic ambiguity may contribute to misdiagnosis and undertreatment of CN, particularly in its early stages [2, 4, 5, 8].

As diabetes prevalence rises, CN's burden is expected to grow, underscoring the need for improved screening [2, 3, 4, 5]. Diagnosis relies primarily on clinical examination and imaging (radiographs, magnetic resonance imaging [MRI]) [2, 3, 8], though early structural changes may be subtle and advanced imaging access can be limited [5, 8]. Gait analysis offers a non‐invasive means of assessing joint function and abnormal loading patterns that may inform offloading and rehabilitation strategies [9, 10]. Prior studies have used gait analysis to evaluate the effects of diabetic neuropathy on joint kinematics and ground reaction forces, reporting slower gait speed, shorter step length, reduced ankle and knee motion, diminished plantarflexion moments, and altered load transfer from hindfoot to forefoot in affected individuals [11, 12, 13, 14]. Increased midfoot plantar pressures and abnormal plantar pressure redistribution have similarly been described in CN specifically [15, 16, 17]. These changes are believed to contribute to progressive deformity, impaired functional mobility, and increased ulceration risk by concentrating repetitive loading across vulnerable regions of the foot. However, many existing protocols rely on expensive or complex three‐dimensional motion capture equipment and specialized multi‐segment foot models that limit routine clinic accessibility [18, 19, 20, 21].

It remains unclear whether a simplified, clinic‐based integrated gait analysis can detect gait kinematic and plantar pressure patterns comparable to those reported using more complex gait analysis methods in patients with CN. This gap is clinically relevant, as early CN changes are frequently missed in outpatient settings lacking advanced gait laboratories, underscoring the value of an affordable, clinic‐accessible protocol that can support patient monitoring, offloading, and rehabilitation planning. The primary aim of this study was to evaluate the utility of an affordable, clinic‐accessible two‐dimensional gait and plantar pressure analysis set‐up in identifying characteristic gait and plantar pressure patterns in CN. CN has been reported to involve bilateral, though often asymmetric, disease [2], and plantar pressure specifically has not shown significant side‐to‐side differences [15]; whether this bilateral pattern extends to gait kinematics has not been established. Our secondary aim was therefore to evaluate differences both between affected and contralateral limbs and between contralateral limbs and Healthy controls. We hypothesized that this clinic‐based approach would detect increased midfoot plantar pressures and altered gait kinematics in both affected and contralateral limbs compared with Healthy controls, and that differences would also be present between affected and contralateral limbs. This study is presented as an initial pilot investigation intended to inform the design of subsequent larger‐scale studies.

## 2 Methods

### 2.1 Study Design

This cross‐sectional observational study was conducted at a single tertiary care center between July 2024 and December 2024 and was approved by the institutional review board (IRB Protocol #00021310). As a pilot and feasibility investigation, the primary aim of the study was to evaluate whether our proposed approach could detect predictable, known biomechanical differences in plantar pressures and gait kinematics between patients with CN and Healthy controls, establishing proof‐of‐concept for this approach rather than providing a definitively powered comparative analysis. Our secondary aim was to examine these differences between affected and contralateral limbs and between contralateral limbs and Healthy controls in patients with CN. Informed consent was obtained from all participants.

### 2.2 Subject Selection

Potential participants for the CN group were identified by study‐affiliated providers, and eligibility was confirmed through a preliminary chart review of the electronic medical record. The following screening, recruitment was conducted by IRB‐approved research staff either by telephone or after the patient's clinic visit. All participants were required to be ≥ 21 years of age.

Inclusion criteria for the CN group included a documented diagnosis of diabetic neuropathic arthropathy verified using ICD‐10 codes [E10.610, E11.610, or E13.610]. All diabetes subtypes (Type 1, Type 2, and other specified) were eligible, as CN arises from underlying peripheral neuropathy irrespective of subtype, and has been well documented in patients with Type 1 diabetes specifically [22]. Alternatively, patients with a diagnosis of the Charcot joint (ICD‐10 codes [M14.671, M14.672, or M14.679]) and a separate diagnosis of diabetes mellitus were also eligible. Inclusion criteria for the control (“Healthy”) cohort included absence of diabetes, neuropathy, or known gait‐affecting conditions.

Exclusion criteria for all participants included active plantar foot ulcers or wounds, inability to bear weight on a lower extremity, presence of a lower extremity prosthesis, and bilateral CN—given that both limbs are affected, precluding a within‐patient comparison. Inflammatory and degenerative musculoskeletal conditions (e.g., osteoarthritis, rheumatoid arthritis) affecting joint range of motion (ROM) were screened for and excluded in the Healthy cohort but were not systematically assessed in the CN cohort. All subjects were enrolled consecutively.

### 2.3 Experimental Procedures

Demographic data (body mass index [BMI], age, sex, and relevant diagnoses) were collected for both cohorts via a brief participant survey.

Gait and plantar pressure data were collected in a standard clinic immediately following each participant's appointment, using a portable plantar pressure measurement system and video‐based motion capture tools. Subjects walked on a one‐meter‐long treadmill wearing color‐coded markers and diabetic slippers equipped with pressure sensor inserts. Each subject completed a series of walking trials at a controlled speed of 1.0 mph, during which plantar pressures and two‐dimensional sagittal‐plane motion were captured. Data collection was only performed once the subject was walking at the appropriate speed to avoid including acceleration and deceleration.

### 2.4 Plantar Pressure Measurements

Participants were fitted with an in‐shoe plantar pressure measurement system (Tekscan Inc. Norwood, MA) consisting of a matrix of 64 individual sensors with a spatial resolution of 3.9 sensing points per cm^2^. Sensors were calibrated and zeroed according to the manufacturer prior to data collection. Plantar pressure data were sampled at 240 Hz, with an average of 8 steps per trial. Data collection was repeated to capture measurements from both lower extremities.

Peak plantar pressures were calculated for each step and normalized to body weight to account for inter‐individual differences in loading. Using the accompanying software, the pressure matrix was discretized into predefined regions of interest (ROIs): forefoot, metatarsal, midfoot, hindfoot and total foot area, as regional plantar pressure analysis lacks a single standardized masking convention [23]. Our model was modified from the five‐region scheme described by Cavanagh and colleagues for plantar pressure assessment in diabetics, balancing clinical accessibility with regional specificity (Figure 1) [24]. The metatarsal region was isolated from the forefoot given the established clinical significance of the metatarsal heads, under which approximately half of diabetic foot ulcers develop [25] and where pressure is driven by local deformity mechanisms distinct from those at the toes [26]. This approach is consistent with comparable diabetic and CN pressure studies [27, 28, 29, 30, 31]. Analysis was limited to the first four ROIs to better characterize regional differences between cohorts.

**FIGURE 1:**
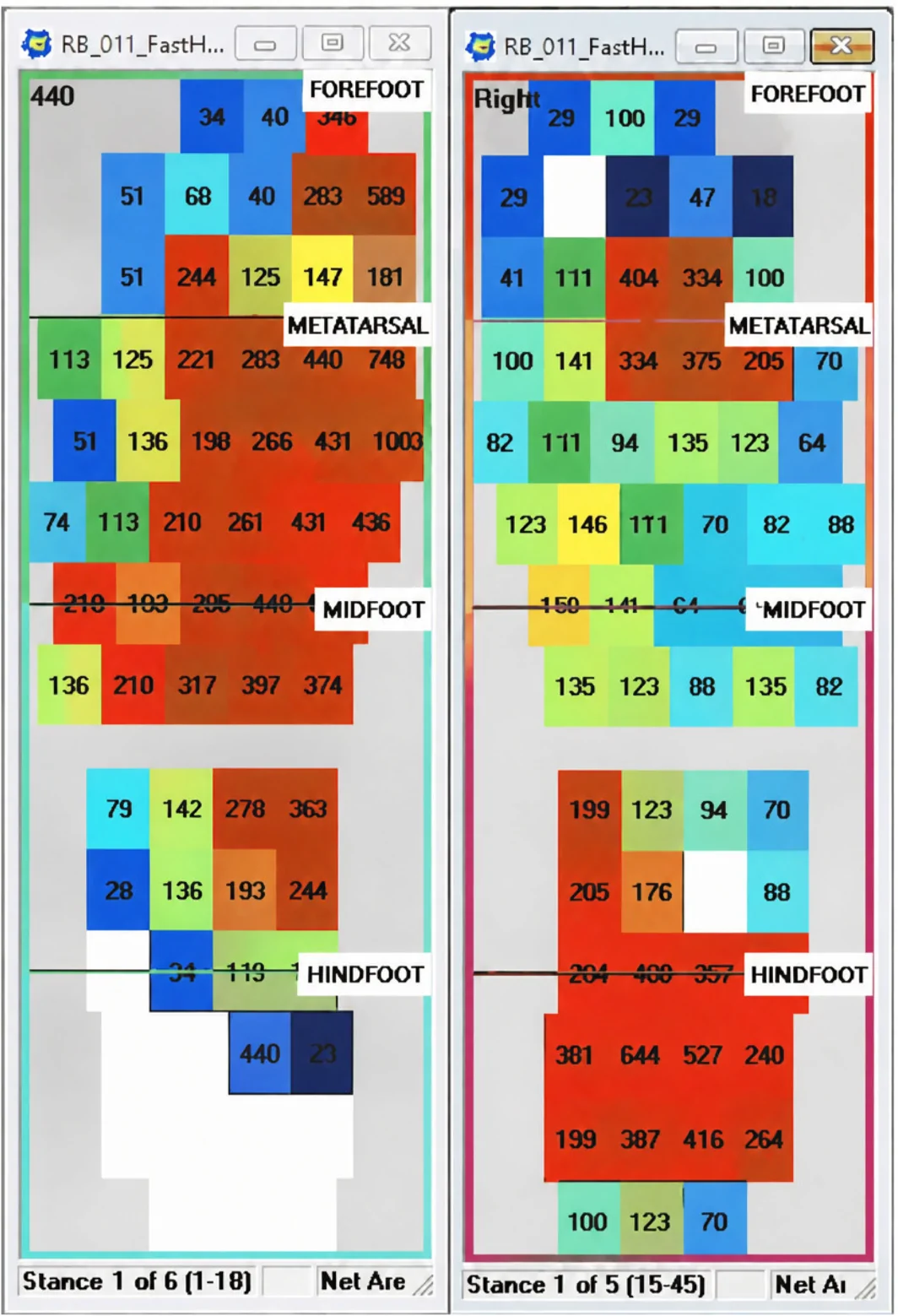
Regional plantar pressure masking scheme. Representative left (L) and right (R) foot plantar pressure distributions divided into the four regions of interest (ROIs) used for statistical analysis: forefoot, metatarsal, midfoot, and hindfoot. Cell values represent raw sensor pressure readings from a representative step. This four‐region scheme was loosely adapted from the five‐region convention described by Bennetts et al. [23], with the metatarsal isolated as a distinct zone given its clinical significance in diabetic and Charcot foot pathology [24, 25].

### 2.5 Motion‐Capture Kinematic Gait Analysis

Two‐dimensional gait kinematics were assessed using a single‐segment foot model to ensure that the reported ankle angle reflects the orientation of the entire foot‐ankle complex relative to the shank rather than isolated ankle joint motion independent of the foot. Additionally, this improves clinical accessibility as two‐dimensional video‐based motion capture has demonstrated acceptable reliability and validity for sagittal‐plane joint angle measurement relative to three‐dimensional motion capture, particularly for larger joints such as the knee and hip [19]. Color‐coded reflective markers were placed over standardized black pants at key anatomical landmarks: the greater trochanter, lateral femoral condyle, lateral malleolus, and distal fifth metatarsal (Figure 2A). Video recordings were acquired at 24 frames per second and 4k resolution. Camera position was standardized across all trials relative to the treadmill, with fixed posterior, lateral, and vertical distances to ensure consistent capture of sagittal‐plane motion.

**FIGURE 2:**
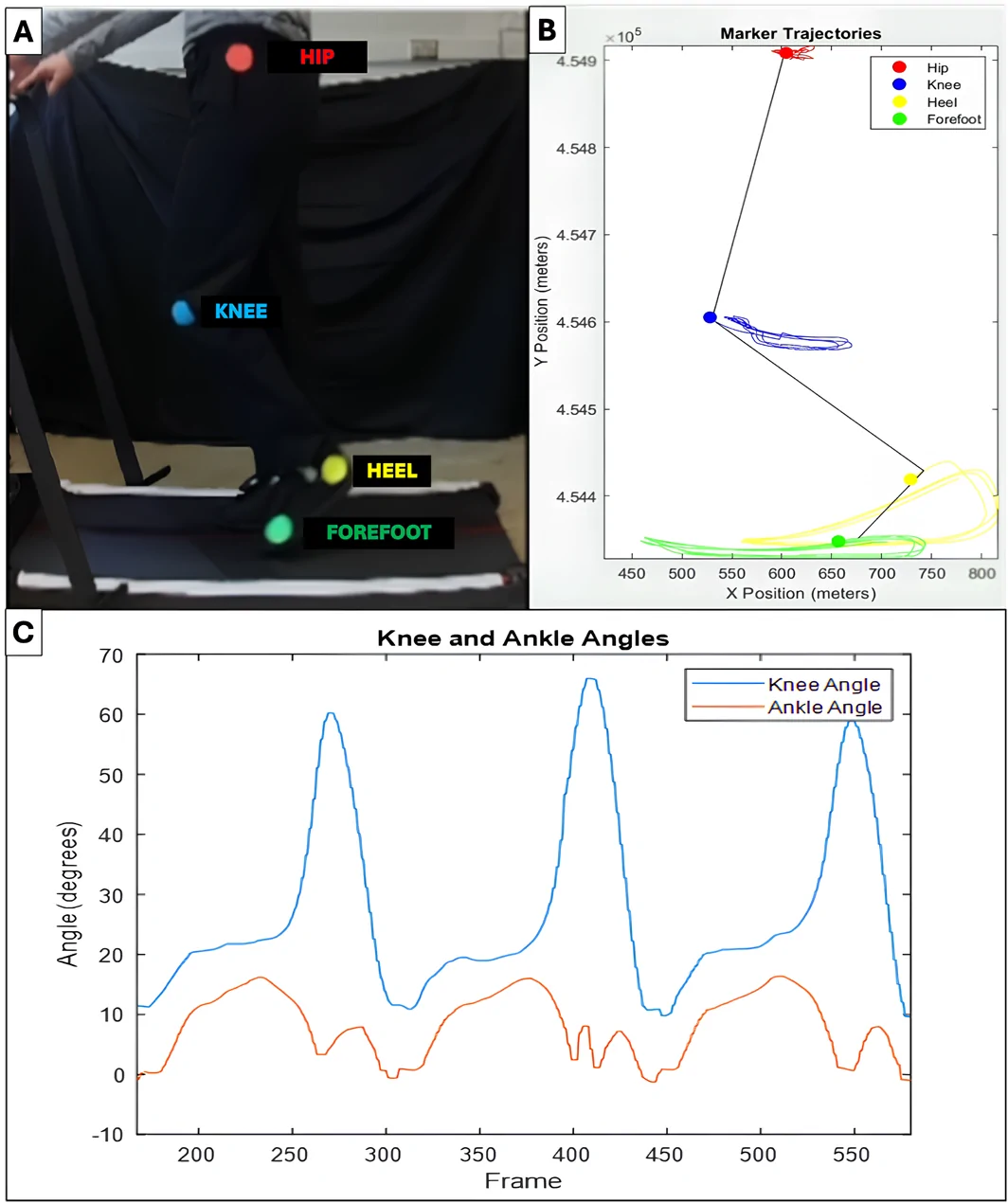
Motion capture setup and representative kinematic data. (A) Treadmill gait setup with color‐coded markers over standardized clothing at the hip (red), knee (blue), heel (yellow), and forefoot (green), tracking lower‐extremity motion. (B) Representative hip, knee, heel, and forefoot trajectories (sagittal‐plane X/Y position, meters) across successive gait cycles generated in MATLAB (MathWorks, Natick, MA). (C) Representative knee and ankle angles (degrees) across two gait cycles (frame number) calculated from marker trajectories in the MATLAB program.

Custom MATLAB (MathWorks, Natick, MA) was used to identify and track marker centroids across frames (Figure 2A,B). Marker trajectories were used to generate joint angle‐time profiles and visualizations of thigh, shank and foot segment motion throughout the gait cycle (Figure 2B). Knee and ankle joint angles were calculated using the angular orientation of adjacent limb segments, and joint angles were plotted over time (Figure 2C).

Kinematic variables analyzed included segment lengths, maximum joint angles, and joint range of motion at the hip, knee, and ankle. In the Healthy cohort, right‐limb kinematic data were unavailable due to data loss during processing, restricting kinematic analyses to the left limb; plantar pressure analyses were also restricted to the left limb for methodological consistency and to avoid statistical non‐independence between limbs (see Limitations). We acknowledge that this differs from the CN cohort, in which the affected limb, regardless of laterality, was used, and address this further in the Limitations.

### 2.6 Outcome Measures

Plantar pressure data were collected across multiple steps during walking. For each step, peak plantar pressure (kPa) was calculated on a per–unit area basis. Step‐level peak pressures were averaged within each predefined region of interest. The median of these region‐specific averaged peak pressures was used for statistical comparisons.

For gait kinematics, outcome measures included the average joint range of motion of the hip, knee, and ankle over recorded step cycles.

Six primary comparisons were performed—affected‐versus‐healthy, contralateral‐versus‐healthy, and affected‐versus‐contralateral limbs—each evaluated separately for plantar pressure and kinematic outcomes.

### 2.7 Statistical Analysis

Nonparametric Mann–Whitney U tests were used to compare demographic, plantar pressure, and kinematic outcomes between the CN and Healthy groups, given unequal sample sizes and non‐normal data distributions. Statistical significance was set at *p* < 0.05. Group differences were interpreted using median values, with interquartile ranges (IQRs) and Mann–Whitney *U*‐statistics also reported.

Within the CN cohort, comparisons between affected and contralateral limbs were performed using paired t‐tests when Shapiro–Wilk testing supported an assumption of normality of the paired differences, and Wilcoxon signed‐rank tests otherwise. Given the number of comparisons performed across plantar pressure and kinematic outcomes (*m* = 21 tests total), *p*‐values were corrected for multiple comparisons using the Benjamini‐Hochberg procedure to control the false discovery rate; Benjamini‐Hochberg‐adjusted *p*‐values are reported as the primary result, alongside uncorrected *p*‐values.

## 3 Results

### 3.1 Demographic Data

CN patients (median age 57.0 years, IQR 56.0–69.0; median BMI 34.22 kg/m^2^, IQR 31.24–39.67) were significantly older (Mann–Whitney *U* = 94.0, *p* < 0.001) and had significantly higher BMI (*U* = 93.0, *p* = 0.001) than Healthy controls (median age 25 years, IQR 23–27; median BMI 26.6 kg/m^2^, IQR 25.33–29.30) (Table 1). Within the CN cohort, 89% (8/9) had a documented diagnosis of Type 2 diabetes mellitus, with a median HbA1c of 6.80% (IQR 6.00–7.10), 11% (1/9) had a history of peripheral artery disease, and 22% (2/9) had a history of osteomyelitis (Table 1).

**TABLE 1:** Demographic and clinical characteristics (*n* = 9 CN, *n* = 11 healthy).

Variable	CN (*n* = 9)	Healthy (*n* = 11)	*p*‐value	Notes
General Demographics				
*n* (patients)	9	11		
Age, median [IQR]	57.0 [56.0–69.0]	25 [23–27]	0.0008	Mann‐Whitney U = 94.0
Female, %	56% (5/9)	54% (6/11)	1	
BMI, median [IQR]	34.22 [31.24–39.67]	26.6 [25.33–29.30]	0.0011	Mann‐Whitney U = 93.0
Diabetes/Metabolic				
T2DM, %	89% (8/9)	0% (exclusion criterion)	—	Not compared—Healthy exclusion criteria
HbA1c, median [IQR]	6.80 [6.00–7.10]	Not assessed	—	HbA1c not tested in Healthy cohort
Systemic comorbidities				
History of PAD, %	11% (1/9)	0% (0/11)	—	Not compared—none reported in Healthy cohort
CKD/Dialysis, %	33% (3/9)	0% (0/11)	—	Not compared—none reported in Healthy cohort
Hyperparathyroidism, %	33% (3/9)	0% (0/11)	—	Not compared—none reported in Healthy cohort
Musculoskeletal comorbidities				
Inflammatory joint disease (formal dx), %	33% (3/9)	0% (0/11)	—	Excluded by design in Healthy cohort; not systematically screened in CN cohort (see Limitations)
CN‐specific disease characteristics				
CN duration, yrs, median [known cases]	3.0 (*n* = 7 known)	N/A	—	Not applicable to healthy cohort
Anatomic pattern of CN involvement	Midfoot only: 2Midfoot + ankle: 4Midfoot + forefoot: 1Forefoot + midfoot + ankle: 1 (*n* = 8 known)	N/A	—	Not applicable to healthy cohort
History of osteomyelitis, %	22% (2/9)	0% (0/11)	—	Not compared—none reported in Healthy cohort
History of prior ulceration, %	56% (5/9)	0% (0/11)	—	Not compared—none reported in Healthy cohort
Prior foot/ankle surgery, %	89% (8/9)	0% (0/11)	—	Not compared—none reported in Healthy cohort

*No*te: CN, *n* = 9 patients. Healthy, *n* = 11 participants. *“N/A” indicates the variable does not conceptually apply to the Healthy cohort*. *p* value “—” indicates the comparison was not performed because the group difference is guaranteed by Healthy cohort exclusion/screening criteria rather than reflecting a new finding.

Abbreviations: BMI, body mass index; CN, charcot neuroarthropathy; HbA1c, glycated hemoglobin; PAD, peripheral artery disease. T2DM, type 2 diabetes mellitus.

### 3.2 Plantar Pressure Data

Compared with Healthy controls, affected limbs demonstrated significantly lower hindfoot (*U* = 16.0, adjusted *p* = 0.043), metatarsal (*U* = 9.0, adjusted *p* = 0.017), and forefoot pressures (*U* = 15.0, adjusted *p* = 0.041), and significantly higher midfoot pressure (median 53.0% BW, IQR 32.0–72.0) than controls (median 21.0% BW, IQR 18.0–35.0; *U* = 84.0, adjusted *p* = 0.041) (Table 2). Contralateral limbs did not differ significantly from Healthy controls in any region after correction for multiple comparisons (adjusted *p* > 0.05 for all four regions), although hindfoot pressure showed a trend prior to correction (raw *p* = 0.023). Plantar pressure distributions for affected, contralateral, and Healthy limbs are shown in Figure 3.

**TABLE 2:** Averaged peak plantar pressures: median (IQR), *U*, and *p*‐values for listed ROIs (hindfoot, midfoot, metatarsals, and forefoot) in CN and Healthy groups^a^.

Region of interest	Affected	Contralateral	Healthy	Affected vs. healthy		Contralateral vs. healthy		Affected vs. contralateral	
(BW%)	Median [IQR]	Median [IQR]	Median [IQR]	(Raw *p*)	(BH‐adj *p*)	(Raw *p*)	(BH‐adj *p*)	(Raw *p*)	(BH‐adj *p*)
Hindfoot	0.21 [0.14–0.48]	0.27 [0.21–0.66]	0.70 [0.58–0.79]	0.0122	**0.0426**	0.0227	0.0595	0.8245	0.9113
Midfoot	0.53 [0.32–0.72]	0.33 [0.22–0.52]	0.21 [0.18–0.35]	0.0098	**0.0411**	0.2241	0.3362	0.2043	0.3301
Metatarsals	0.12 [0.05–0.46]	0.61 [0.29–0.66]	1.05 [0.72–1.24]	0.0024	**0.0166**	0.0806	0.1692	0.0218	0.0595
Forefoot	0.02 [0.01–0.07]	0.19 [0.04–0.41]	0.27 [0.09–0.45]	0.0098	**0.0411**	0.704	0.8214	0.0791	0.1692

*No*te: Individual peak plantar pressure values were normalized to body weight. Regions of interest (ROIs) were based off of a four‐region modified from the five‐region scheme described by Cavanagh and Ulbrecht [24]. CN, affected, *n* = 9, Contralateral, *n* = 9. Healthy, *n* = 11. Bold red = BH‐adjusted *p* < 0.05.

Abbreviations: CN, charcot neuroarthropathy; ROI, region of interest.

^a^ *p*‐values computed via Mann‐Whitney *U* (between‐group) or paired t‐test/Wilcoxon signed‐rank (affected vs contralateral, matched within‐patient), corrected for multiple comparisons (*m* = 21) using Benjamini‐Hochberg FDR.

**FIGURE 3:**
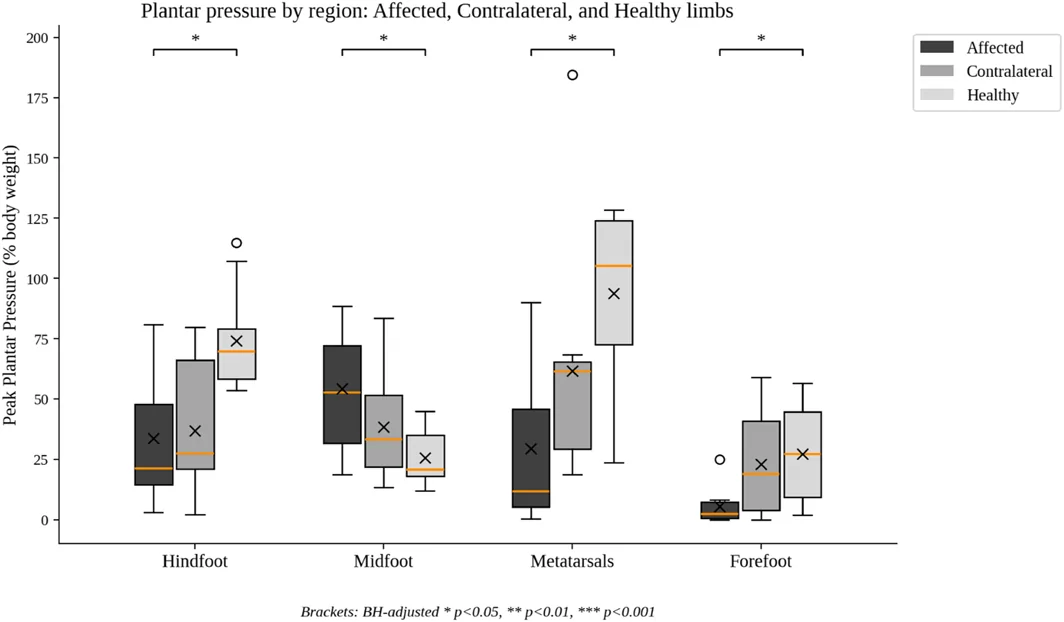
Peak plantar pressure by anatomic region of interest: CN‐affected, contralateral, and Healthy control limbs. Box‐and‐whisker plots display peak plantar pressure (% body weight) at four predefined regions of interest (hindfoot, midfoot, metatarsals, forefoot) for CN‐affected limbs (*n* = 9), contralateral limbs (*n* = 9), and the left limb of Healthy controls (*n* = 11). Box hinges represent the 25th and 75th percentiles, the horizontal line within each box indicates the median, the X marker indicates the mean, whiskers extend to the 10th and 90th percentiles, and open circles represent values outside this range. Brackets denote statistically significant affected‐versus‐Healthy comparisons after Benjamini–Hochberg correction across all 21 statistical tests (*p < 0.05); no other pairwise comparisons within these regions reached significance. Affected limbs showed significantly elevated midfoot pressure relative to Healthy controls, consistent with midfoot structural collapse, whereas hindfoot, metatarsal, and forefoot pressures were significantly reduced in affected limbs relative to Healthy controls.

No significant differences were detected between affected and contralateral CN limbs in any plantar pressure region after correction for multiple comparisons (adjusted *p* > 0.05 for all four regions), although the metatarsal region showed a trend prior to correction (raw *p* = 0.022) that did not survive Benjamini‐Hochberg adjustment (adjusted *p* = 0.060).

### 3.3 Kinematic Data

Knee range of motion was significantly reduced in both affected limbs (median 44.85°, IQR 38.82–48.13; *U* = 1.0, adjusted *p* = 0.006) and contralateral limbs (median 48.88°, IQR 46.39–52.48; *U* = 6.5, adjusted *p* = 0.013) compared with Healthy controls (median 58.77°, IQR 56.50–60.61), with no significant difference between affected and contralateral limbs (adjusted *p* = 0.207). No significant differences were observed at the hip or ankle for any comparison (adjusted *p* > 0.05) (Table 3). Knee, hip, and ankle range‐of‐motion data are shown in Figure 4.

**TABLE 3:** Kinematic outcomes: median (IQR), *U*, and *p*‐values for hip, knee and ankle joint angles observed in the CN and Healthy groups^a^.

Joint (Degrees)	Affected	Contralateral	Healthy	Affected vs. healthy		Contralateral vs. healthy		Affected vs. contralateral	
	Median [IQR]	Median [IQR]	Median [IQR]	(Raw *p*)	(BH‐adj *p*>)	(Raw *p*)	(BH‐adj *p*)	(Raw *p*)	(BH‐adj *p*)
Hip	32.83 [30.52–36.75]	32.18 [29.47–38.26]	31.92 [28.20–34.70]	0.8792	0.9181	0.6485	0.8011	0.9181	0.9181
Knee	44.85 [38.82–48.13]	48.88 [46.39–52.48]	58.77 [56.50–60.61]	0.0003	**0.0056**	0.0012	0.013	0.1084	0.207
Ankle	23.11 [13.59–30.84]	16.02 [14.62–20.33]	19.57 [14.84–23.48]	0.5949	0.7807	0.4033	0.5646	0.1614	0.2824

*No*te: CN, affected, *n* = 9, Contralateral, *n* = 9. Healthy, *n* = 11. Bold red = BH‐adjusted *p* < 0.05.

Abbreviations: CN, charcot neuroarthropathy; ROM, range of motion (degrees).

^a^ *p*‐values computed via Mann‐Whitney *U* (between‐group) or paired t‐test/Wilcoxon signed‐rank (affected vs contralateral, matched within‐patient), corrected for multiple comparisons (*m* = 21) using Benjamini‐Hochberg FDR.

**FIGURE 4:**
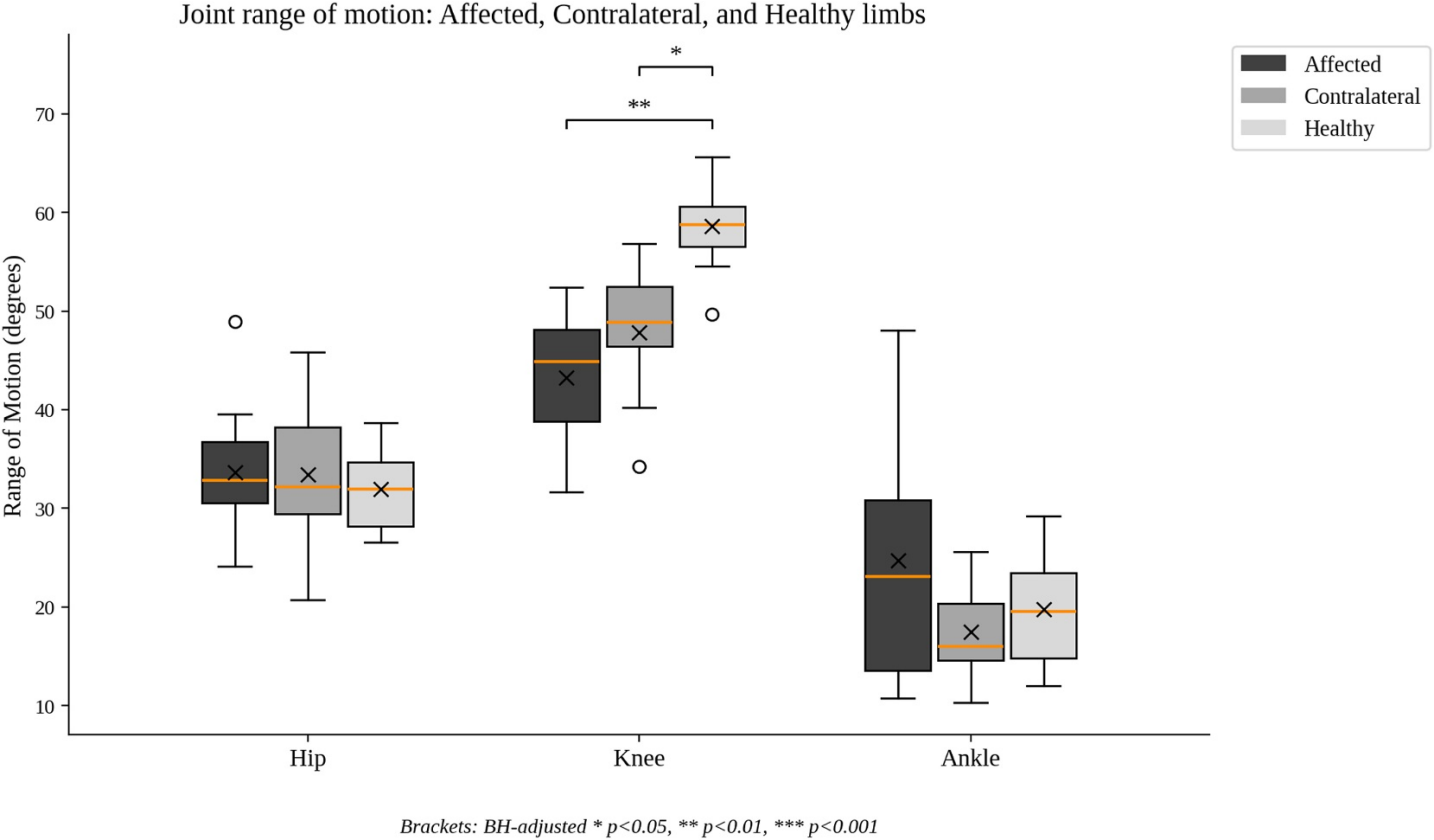
Sagittal‐plane joint range of motion during gait: CN‐affected, contralateral, and Healthy control limbs. Box‐and‐whisker plots display 2D sagittal‐plane range of motion (degrees) at the hip, knee, and ankle for CN‐affected limbs (*n* = 9), contralateral limbs (*n* = 9), and the left limb of Healthy controls (*n* = 11). Box hinges represent the 25th and 75th percentiles, the horizontal line within each box indicates the median, the X marker indicates the mean, whiskers extend to the 10th and 90th percentiles, and open circles represent values outside this range. Brackets denote statistically significant comparisons after Benjamini–Hochberg correction across all 21 statistical tests (*p < 0.05, **p < 0.01); comparisons not shown were not statistically significant. Knee range of motion was significantly reduced in both affected and contralateral limbs relative to Healthy controls, while no significant differences were observed at the hip or ankle.

## 4 Discussion

This study evaluated the feasibility of a clinic‐based integrated gait and plantar pressure assessment for identifying biomechanical alterations in patients with Charcot neuroarthropathy (CN). Compared with Healthy controls, CN patients demonstrated increased midfoot plantar pressures, reduced plantar pressures in the forefoot, metatarsal, and hindfoot regions, and decreased knee range of motion.

Midfoot plantar pressures were significantly higher in the CN cohort compared with controls (adjusted *p* = 0.041). In the normal gait cycle, plantar loading progresses from the hindfoot at heel strike to the forefoot during terminal stance, as ankle plantarflexion facilitates forward propulsion and prepares the limb for toe‐off [2, 4, 11]. In patients with diabetic neuropathy, nonenzymatic glycation and soft tissue stiffening impair the windlass mechanism and reduce plantarflexion moments, disrupting this normal load transfer pattern [2, 11, 12]. As a result, force progression toward the forefoot is diminished and loading may remain concentrated at the midfoot. This may reflect early biomechanical changes preceding or accompanying the rocker‐bottom deformity seen in advanced CN [4, 5]. These also support the hyperemia‐driven pathway described in the Introduction. Direct structural assessment of the plantar aponeurosis and ankle‐foot unit was beyond this study's scope and warrants future investigation [32].

While prior studies in diabetic neuropathy and CN have reported elevated forefoot and metatarsal pressures in addition to midfoot changes [4, 15], we observed reduced plantar loading in the hindfoot, forefoot, and metatarsal regions, consistent with the findings of Keukenkamp et al. [16] (Figure 3). Differences in disease stage, deformity severity, or patient characteristics may explain this variation.

Despite reproducing the expected midfoot pressures characteristic of CN deformity, we did not detect significant differences in peak plantar pressures between the affected and contralateral limbs of CN patients after correction for multiple comparisons (adjusted *p* > 0.05 for all four regions). Similar findings have been reported by Armstrong et al. [15]. Contralateral limbs also did not differ significantly from Healthy controls in plantar pressure (adjusted *p* > 0.05 for all four regions), suggesting that the contralateral limb's pressure profile may genuinely fall between the affected and Healthy groups, rather than the null result simply reflecting limited statistical power. In contrast, knee range of motion was significantly reduced in both affected and contralateral limbs relative to Healthy controls, with no significant difference between affected and contralateral limbs themselves. This pattern, evident in kinematics but not plantar pressure, is consistent with CN's recognized tendency toward bilateral, though often asymmetric, involvement [2], and suggests that gait kinematic changes may extend to the contralateral limb even when compensatory pressure redistribution does not or does so to a lesser degree. Contralateral plantar pressures, while not significantly different from either the affected limb or Healthy controls, fell between the two. These observations raise the possibility that gait kinematic changes become detectable before plantar pressure changes reach statistical significance, a hypothesis warranting further investigation in larger cohorts.

Knee range of motion was reduced in both affected and contralateral CN limbs compared with Healthy controls (Figure 4), a bilateral pattern that was not observed for plantar pressure, suggesting bilateral biomechanical involvement in this cohort. Prior studies have demonstrated slower gait, shorter step length, reduced ankle and knee motion, and diminished plantarflexion moments in individuals with diabetic neuropathy [11, 12, 13, 14]. Reduced push‐off power and impaired load transfer from hindfoot to forefoot may contribute to proximal compensatory strategies, including decreased knee movement [2, 11, 13]. Two of the nine CN patients had documented knee‐related inflammatory or degenerative joint disease (Table 1). Osteoarthritis and peripheral neuropathy severity were not formally assessed and cannot be isolated as contributors to this finding (see Limitations). Although we anticipated reduced ankle plantarflexion given the close biomechanical relationship between the ankle and midfoot, total ankle range of motion was preserved in the CN cohort. This unexpected finding may reflect compensatory ankle dorsiflexion due to foot deformity and loss of tissue elasticity [4, 11, 12, 13], or limitations of our two‐dimensional single‐segment foot model in isolating hindfoot‐specific motion contributions to total ankle excursion. Multi‐segment foot models in larger cohorts may help clarify this relationship.

This study has several limitations that may affect the strength and scope of our conclusions.

### 4.1 Selection and Sample Characteristics

Our CN participants were recruited from a single tertiary care center, potentially introducing selection bias; whether this protocol performs comparably across different clinical settings and patient populations remains to be tested. The sample size was modest (9 CN patients, 11 Healthy controls), reducing statistical power and preventing further subgroup analyses. CN is a comparatively rare complication even among a growing diabetic population, and comparably sized cohorts are consistent with other biomechanical studies of this population (e.g., a 2024 study evaluating offloading footwear in patients with Charcot neuropathy enrolled 15 participants [27]). This study's protocol further narrowed the eligible population, excluding patients with active ulceration, lower‐extremity amputation or prosthesis, or inability to safely ambulate on a treadmill. Whether this protocol performs comparably in these more complicated CN presentations remains untested and is an important direction for future work. Disease duration and CN stage (e.g., acute vs. chronic) were not systematically documented or used as inclusion criteria and may represent a source of heterogeneity in our findings; we did not retrospectively assign disease stage as inconsistent clinical documentation would have introduced interrater variability. Formal metabolic syndrome, prediabetes status, and inflammatory or degenerative musculoskeletal conditions (e.g., osteoarthritis) were not systematically assessed in the CN cohort beyond the BMI, HbA1c, and diabetes status reported in Table 1. CN patients were significantly older and had significantly higher BMI than Healthy controls (Table 1), which may have introduced confounding related to age‐ and weight‐dependent gait differences; neuropathy severity was not formally assessed, limiting our ability to associate gait alterations with the degree of peripheral neuropathy.

### 4.2 Measurement and Protocol Design

Baseline (static) ankle range of motion was not formally assessed prior to gait analysis, limiting our ability to distinguish dynamic compensatory changes from pre‐existing static restrictions. Reflective markers were placed over standardized clothing rather than directly on the skin, which may introduce soft‐tissue and fabric‐related artifact affecting the precision of kinematic measurements. Kinematic and plantar pressure analyses in the Healthy cohort were restricted to the left limb, despite bilateral limb use in the CN cohort, to maintain consistency in our data analysis and statistical interpretation. This is consistent with methodological recommendations for plantar pressure data [33] and with precedent in comparable gait kinematics research supporting single‐limb analysis under high inter‐limb correlation [34]. Regardless, this combination of data unavailability and deliberate restriction may have influenced group comparisons relative to the affected or contralateral limb used in CN patients. The single‐segment foot model used here, chosen for clinical feasibility over more detailed but equipment‐intensive multi‐segment models [4, 10, 20, 35], detected clinically meaningful differences in plantar pressure and kinematics, supporting its potential integration into outpatient settings. However, our claims are limited to detecting characteristic directional patterns of gait and pressure alteration, rather than detailed information obtained with three‐dimensional systems, such as out‐of‐plane motion, rotational components, and comprehensive multi‐segment foot motion.

Our regional plantar pressure masking scheme did not distinguish medial from lateral forefoot loading; prior work has shown that medial‐lateral pressure distribution differs between diabetic/neuropathic and healthy populations [26], and this distinction may represent a clinically meaningful source of variation not captured by our analysis.

### 4.3 Data Analysis

We performed six comparisons and applied a Benjamini‐Hochberg false discovery rate correction [36] across all resulting tests (*m* = 21) to account for multiple comparisons; both raw and corrected *p*‐values are reported, and findings that did not survive correction are interpreted with appropriate caution.

## 5 Conclusion

As a pilot and feasibility investigation, this study suggests that a clinic‐based two‐dimensional gait and plantar pressure analysis protocol can detect measurable differences in joint kinematics and plantar pressure patterns in patients with CN. Elevated midfoot pressures, reduced forefoot, metatarsal, and hindfoot pressures, plus diminished knee ROM in both the affected and contralateral limbs, are consistent with biomechanical alterations associated with CN, and support bilateral involvement in this population. These findings support the potential role of integrated gait analysis as a non‐invasive adjunct to clinical evaluation, with possible applications in guiding offloading strategies and therapeutic decision‐making. Future research should further refine this approach and evaluate how it relates to disease stage, radiographic data and response to intervention to better understand its potential role in clinical assessment and monitoring.

## Author Contributions

**Irene M. Laleye:** investigation, methodology, data collection, data analysis, writing – original draft. **Zachary A. Koroneos:** conceptualization, methodology, data processing, data analysis, writing – review and editing. **Akhil Bolisetti:** data collection, data processing, writing – review and editing. **Carlie Hernjak:** formal analysis, data processing, data collection. **Titus Richardson:** data collection. **Shelby Alwine:** conceptualization, methodology, resources. **Parker Fox:** data collection, review and editing. **Ashlee MacDonald:** resources, supervision. **Michael A. Aynardi:** conceptualization, resources, supervision.

## Funding

The authors have nothing to report.

## Disclosure

The authors have nothing to report.

## Ethics Statement

All procedures were conducted in accordance with the 1964 Helsinki Declaration and its later amendments. The collection and evaluation of all protected patient health information was performed in a Health Insurance Portability and Accountability Act (HIPAA)—compliant manner.

## Consent

This cross‐sectional observational study was approved by the institutional review board (Protocol #00021310) of a tertiary care academic hospital from July 2024–December 2024. Informed consent was obtained from all study participants.

## Conflicts of Interest

Ashlee MacDonald MD has received an AOFAS Grant that is unrelated to this study.

Michael A. Aynardi MD serves as a consultant for Arthrex, Stryker, and Zimmer. Dr. Aynardi holds leadership roles including Chair of the AOFAS Research Committee, Member of the AOA Membership Committee, and Chair of the PA Ortho Program. Dr. Aynardi also provides expert testimony services.

## Data Availability

The datasets generated and analyzed during the current study are not publicly available due to patient privacy considerations but are available from the corresponding author on reasonable request.
